# Water budgeting in conservation agriculture-based sub-surface drip irrigation in tropical maize using HYDRUS-2D in South Asia

**DOI:** 10.1038/s41598-021-93866-6

**Published:** 2021-08-18

**Authors:** Kiranmoy Patra, C. M. Parihar, H. S. Nayak, Biswajit Rana, V. K. Singh, S. L. Jat, Sanjeev Panwar, M. D. Parihar, L. K. Singh, H. S. Sidhu, B. Gerard, M. L. Jat

**Affiliations:** 1 ICAR-Indian Agricultural Research Institute (IARI), New Delhi, India; 2 ICAR-Indian Institute of Maize Research (IIMR), Unit Office, New Delhi, India; 3 Indian Council of Agricultural Research (ICAR), New Delhi, India; 4 ChouharyCharan Singh Haryana Agriculture University (CCSHAU), Hisar, India; 5 Borlaug Institute for South Asia (BISA)-CIMMYT, Ludhiana, India; 6 International Maize and Wheat Improvement Center (CIMMYT), El-Batan, Texcoco, Mexico; 7 International Maize and Wheat Improvement Center (CIMMYT), New Delhi, India

**Keywords:** Plant sciences, Environmental sciences, Hydrology

## Abstract

In water scarce regions of South Asia, diversification of rice with maize is being advocated towards sustainability of cereal-based cropping systems. Adoption of innovative agronomic management practices, i.e., conservation agriculture (CA) and sub-surface drip irrigation (SSDI) are considered as key strategies for much needed interventions to address the challenges of water scarcity under projected climate change. Benefits from CA and SSDI concerning water economy are well-established, however, information about their complementarity and water budgeting in cereal-based systems are lacking. A field study was conducted with process-based model (HYDRUS-2D) to understand water transport, root water uptake and components of soil water balance in maize grown in rotation with wheat after five years of continuous adoption of conservation agriculture. In this study, altogether eight treatments comprising of 6 CA+ treatments (CA coupled with SSDI); permanent beds using sub-surface drip (PB-SSD) with (WR) and without (WOR) crop residue at different N rates, 0, 120 and 150 kg N ha^−1^ were compared with CA (PB using furrow irrigation-FI with crop residue-120 kg N ha^−1^) and conventional tillage practices (CT) (CT using FI without crop residue-120 kg N ha^−1^). Results showed that the model could simulate the daily changes in profile soil water content with reasonable accuracy in all the treatments. Simulated soil water balance indicated higher cumulative root water uptake (CRWU), lower cumulative evaporation (CE) and higher soil water retention in CA+ (PB-SSD+ crop residue at 150 and 120 kg N ha^−1^) than CA and CT plots. Hydrus-2D model efficiency > 0, RMSE between 0.009–0.026 and R^2^ value between 0.80–0.92 at *P* < 0.01 indicates that the model is performing efficiently. The mean evaporation from CA+ treatments was 10 and 36% less than CA and CT treatments, respectively. On average, CRWU under CA+ treatments were 14–48% higher than FI treatments. The mean cumulative deep drainage in CA+ plots was 80–100 mm less than CA and CT plots. In CA+ based plots significantly higher biomass production and radiation use efficiency were observed with reduced water use than CA and CT. Therefore, the study justifies the water-saving nature of CA+, while maintaining higher productivity and meeting the transpiration demand of crops and halting unnecessary evaporation and deep drainage losses.

## Introduction

In South Asia, rice–wheat (RW) system (13.5 million ha) is the principal determinant of food and livelihood security for billions of people^1,2^. Conventional rice cultivation involves intensive tillage for puddling which incurs huge water loss, deteriorates soil physical health and is a main source of anthropogenic greenhouse gas emmision^3^. Since the early 1970s (Green Revolution era), there has been a steady decline in groundwater table in most of the RW system of North-West (NW) India^4–6^. The rate of decline between 1973 and 2001 was about 0.2 m year^−1^, which got hastened by five-fold (1.0 m year^−1^) between 2000 and 2006^5^. Over-exploitation of the groundwater for rice production is the most likely cause and free electricity for irrigation favours the over withdrawal. In this context, alternate resource use efficient and productive maize production system must be developed and deployed for sustainable food security and protecting ground water resources^7^. Adoption of conservation agriculture (CA), i.e., zero tillage with residue retention and crop diversification had ensured higher productivity and improvement in soil physical health^8,9^. Diversification of rice with C_4_ crops like maize, i.e., maize-wheat system is emerging as a potential alternative to RW system in NW-India. Maize-wheat (MW) system is the third most important cropping system (~ 1.86 Mha) and has the potential to expand under the emerging water crisis in the Indo Gangetic Plains (IGP)^10^. MW system with CA-based practices has twin benefits of superior food and fodder supply as well as enhanced soil health and water productivity (WP)^11^.

Adoption of micro-irrigation is a promising approach for irrigation water-saving relative to conventional irrigation, i.e., furrow irrigation (FI)^5,12^. Precise water management through drip irrigation has shown numerous benefits in terms of water savings, yield and nutrient use efficiency in horticulture and vegetable crops^13^. To address the water scarcity problem surface drip irrigation has been evaluated as a viable option for the cereals like maize^12,14^, rice^15^ and wheat^16^. However, the adoption of surface drip irrigation in cereal-based systems has remained a cumbersome process all the time, as the laterals get anchored during field operations both in conventional and CA systems. To deal with this bottleneck and for better farmers’ acceptance of micro-irrigation in cereal-based systems, subsurface drip irrigation (SSDI) can be advised.

Compared to surface drip irrigation, SSDI restricts the evaporation losses from the soil surface, facilitates the delivery of water and nutrients directly to the root zone that leads to efficient water use, reduces weed emergence and labor cost and allows seeding with CA-based no-tillage practices^17^. Fertigation through drip irrigation system reduces N losses thereby enhancing the nutrient use efficiency^18^. Numerous studies have reported several benefits from CA and SSDI with respect to the water economy. Therefore, there is an excellent scope to design an efficient irrigation scheme by combining these two emerging and novel technologies viz, CA and SSDI in cereal systems of IGP. To advance further, precise information about the components of soil water balance (SWB) is required. The research was designed as informations about the interaction effect of CA and SSDI on water budgeting in a cereal-based system is rare.

Water budgeting can be done with a detailed field experiment, but requirement of instruments like lysimeter and the cost of the experimentation pose limitation to do so. Alternatively modelling the soil hydrological process with a process-based models can give insight about soil water budgeting. A detailed mathematical simulation can help to design a more efficient irrigation system by knowing the fate of applied irrigation in the soil profile. The soil hydrological process-based model Hydrus-2D^19^ can be used to simulate the two-dimensional water movement using Richard's equation for unsaturated flow in different soil layers. Since the initial introduction of Hydrus, it has been effectively evaluated for SWB^20^, groundwater recharge^21^, and for root water uptake^22^ in unsaturated soil. In earlier studies, a good agreement between observed and simulated values for SWB components had been reported^7,23,24^. Previous research based evidences showed that under drip irrigation, Hydrus-2D was successfully calibrated and validated for SWB^25,26^, crop root water uptake^27,28^, soil wetting pattern^29,30^, irrigation scheduling^31,32^ and to evaluate the nutrient transport and distribution pattern^33,34^. Aggarwal et al.^23^ compared the SWB in the scenario of CA and CT and found a very good agreement between observed and simulated values. Parihar et al*.*^9^ simulated soil water dynamics in maize crop and found that cumulative root water uptake (CRWU) was significantly higher in CA-based zero-till (ZT) plots compared to CT plots.

Although the superiority of CA^+^ (CA coupled with SSDI) towards increased WP and resource use efficiency are being reported recently, globally the components of soil water balance has not been quantified in various CA+-based practices. Consequently, for a better understanding of water dynamics in the soil–plant-atmosphere continuum, the present study was conducted during the 6th year of practicing CA-based SSDI (CA+), CA-based FI and CT with different N doses in maize crop. The main focus of this study was to calibrate and validate the Hydrus-2D model using an inverse modelling approach for simulation of SWB in salutations of three tired agronomic innovations ranging from CT to CA and CA+ under different N levels. It was hypothesized that soil moisture content will be higher and less variable in the effective root zone under CA+ SSDI (CA^+^) practices as compared with furrow irrigated CA and CT.

## Results

### Fractional intercepted photosynthetically active radiation (fIPAR) and partition of actual evapotranspiration

**Figure 1:**
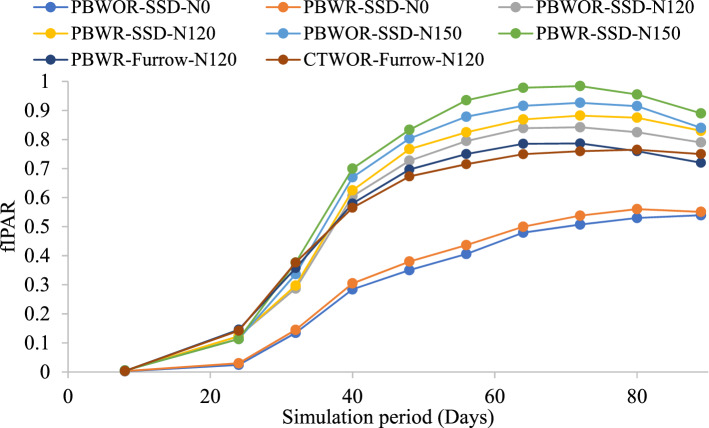
Effect of different tillage, residue, N and irrigation management treatments on fIPAR of maize. fIPAR: Fractional Intercepted Photosynthetically Active Radiation.

During the initial crop phase (1–13 days of simulation; DOS) the fIPAR values were low (0.009–0.047) (Fig. 1). Maximum light interception by crop canopy was observed during 65 DOS (i.e., 72-Days after sowing) in all the treatments except those without N (received no N). During 65 DOS (72-Days after sowing), the highest fIPAR (0.98) was observed in the PBWR-SSD-N150 plots followed by PBWOR-SSD-N150 (0.92). However, CT and CA plots with 120 kg N ha^−1^ application had similar fIPAR throughout the simulation period. On average, adoption of SSDI improved the light interception by 16% over FI during the peak growth period. The fIPAR values were used for partitioning ETc into actual evaporation and actual transpiration. The estimated evaporation and transpiration using Hydrus-2D followed a similar pattern as that of actual evaporation and transpiration.

### Model calibration and validation

**Figure 2:**
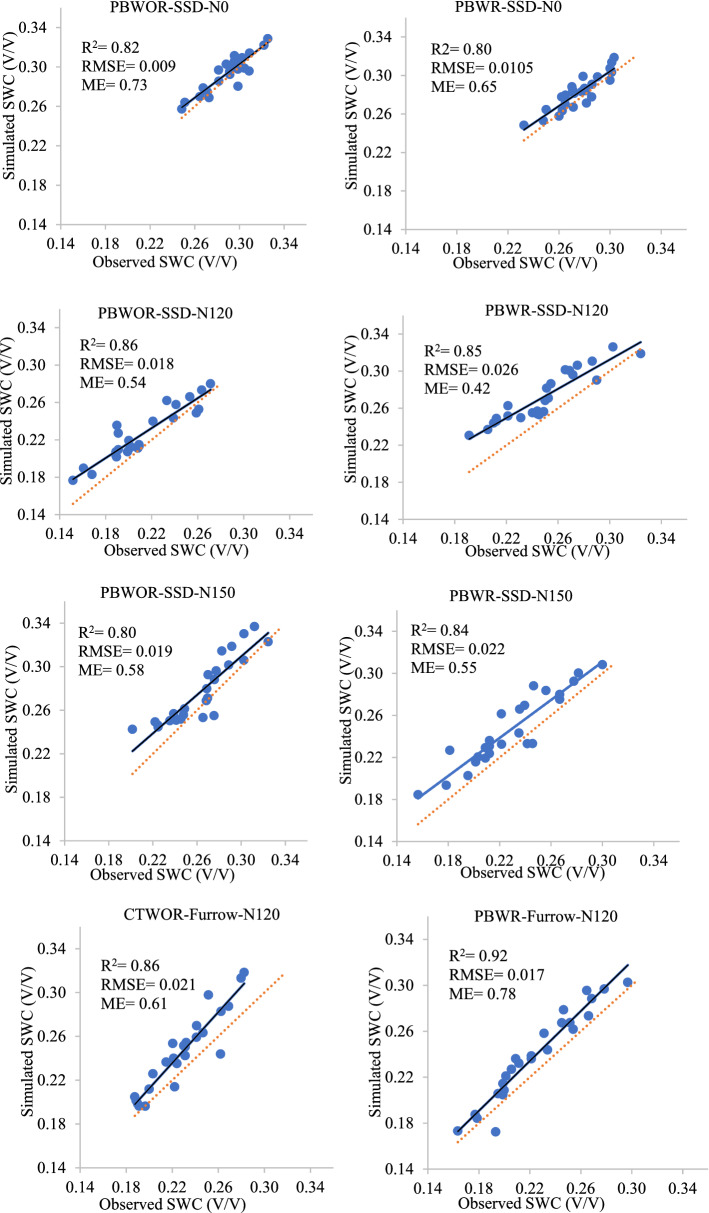
Observed and simulated soil water contents (SWCs) under different tillage, residue, N and irrigation management treatments. RMSE: Root Mean Square Error; ME: Model efficiency. For treatment detail please see Table 4. The continuous line is the relation between observed vs predicted and dotted line is 1:1 line.

The model was well calibrated with the optimized soil hydraulic parameters with R^2^ and RMSE values for all the treatments. The comparison between observed and simulated soil water content along with 1:1 line is depicted in Fig. 2. For all the treatments it was observed that the model over-predicts the moisture content. However, the coefficient of determination value for all treatments lies between 0.80 to 0.92. This indicates that the model could appropriately describe the variation in the soil moisture content. The calculated RMSE value for all the treatments ranged between 0.009 (PBWOR-SSD-N0) to 0.026 (PBWR-SSD-N120). The positive values of ME indicate efficient performance of the model for the entire treatments understudy. Therefore, from the obtained values of the parameters viz, R^2^, RMSE, ME, it can be concluded that HYDRUS-2D model can be used for simulation of soil moisture dynamics effectively for all the set of treatments covering CA+, to CA and conventional practices.

### Soil water balance components

#### Evaporation

**Figure 3:**
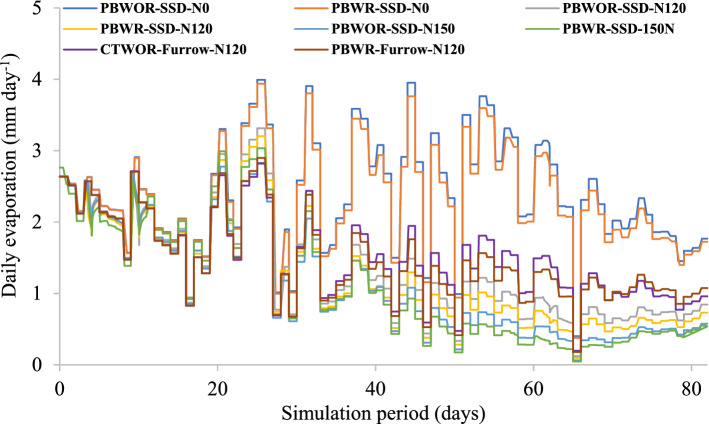
Daily evaporation (mm day^−1^) pattern during the simulation period in maize under different tillage, residue, N and irrigation treatments. For treatment detail please see Table 4.

**Figure 4:**
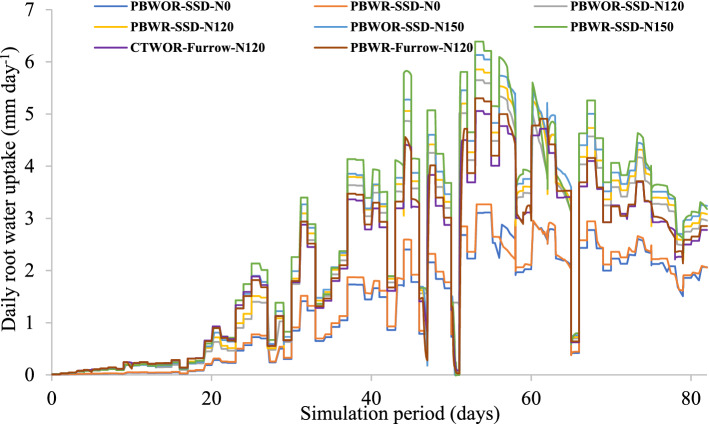
Daily root water uptake (mm day^−1^) pattern during the simulation period in maize under contrasting tillage, residue, N and irrigation treatments. For treatment detail please see Table 4.

The daily evaporation and root water uptake of all treatments are presented in Figs. 3 and 4, respectively. During the initial 20 days of simulation (28 DAS) daily root water uptake from different treatments were negligible (Fig. 4), while daily evaporation ranged between 0.8 to 2.7 mm day^−1^. In treatments without N fertilization (N0), evaporation losses were comparatively higher (1.4–4.0 mm day^−1^) than N-fertilized treatments. After the initial 20 days of simulation, the contrasting effect of CA+, CA and CT along with N doses and irrigation methods on daily evaporation rate became prominent (Fig. 3). The highest cumulative evaporation (195.6 mm) was observed for PBWOR-SSD-N0, while the minimum cumulative evaporation (85.3 mm) was recorded for PBWR-SSD-N150 (Table 1). The cumulative evaporation losses from the CA+ treatments with N, i.e., PBWR-SSD-N150 and PBWR-SSD-N120 were ~ 24% and 10% lower than CA treatment PBWR-Furrow-N120. Similarly, PBWR-SSD-N150 and PBWR-SSD-N120 recorded ~ 36% and 24% less evaporation compared to conventional-CTWOR-Furrow-N120.

**Table 1:** Soil water balance (SWB) components computed by using HYDRUS-2D for simulation period (8–89 DAS) of maize.

Treatments^a^	CRWU (mm)	CE (mm)	CDD (mm)	SWC Initial (mm)	SWC Final (mm)	CVF (mm)	CI (mm)	SWB
PBWOR-SSD-N0	66.0	195.6	245.0	117.0	106.5	100	391.2	− 0.50
PBWR-SSD-N0	73.9	189.2	228.2	111.2	110.4	100	391.2	0.06
PBWOR-SSD-N120	156.2	110.8	232.3	81.6	76.7	100	391.2	− 0.31
PBWR-SSD-N120	170.3	101.5	221.5	98.2	90.1	100	391.2	0.60
PBWOR-SSD-N150	181.4	93.1	224.8	97.1	94.6	100	391.2	− 0.55
PBWR-SSD-N150	208.6	85.3	201.1	78.2	82.9	100	391.2	− 0.86
CTWOR-Furrow-N120	141.1	133.6	298.0	105.6	94.1	0	551.2	− 0.99
PBWR-Furrow-N120	149.6	112.5	302.5	104.3	95.5	0	551.2	− 0.46

CRWU: cumulative root water uptake; CE: cumulative evaporation; CDD: cumulative deep drainage; SWC Initial: Initial (on the first day of simulation) cumulative soil–water content (mm) in soil profile up to 45 cm depth; SWC Final: Total soil water content in 45 cm depth of soil on the last day (82th) of simulation; CVF: cumulative variable flux; CI: cumulative infiltration (rainfall + irrigation) for treatments with furrow irrigation and only rainfall for treatments with subsurface drip irrigation; SWB: soil water balance.

^a^For treatment detail please see Table 4.

#### Root water uptake

The root water uptake during the simulation period followed an opposite trend to that of daily evaporation. The peak root water uptake was commenced during 44th to 55th day of simulation which ranged between 5.8 to 6.4 mm day^−1^ (PBWR-SSD-N150), 5.2 to 6.2 mm day^−1^ (PBWOR-SSD-N150), 4.4 to 5.2 mm day^−1^ (PBWR-Furrow-N120) and 4.3 to 5.0 mm day^−1^ (CTWOR-Furrow-N120), respectively (Fig. 4). N application at 150 kg ha^−1^ and 120 kg ha^−1^ increased the daily root water uptake over N0 (without N application) by ~ 3.3 and 2.6-mm day^−1^, respectively during peak period (Fig. 4) and cumulative root water uptake (CRWU) by 179% and 133%, respectively. Switching from the CA to CA+ (CA + SSDI) increased the CRWU by 14% (PBWR-SSD-N120 vs. PBWR-Furrow-N120). However, CA yielded 21% higher CRWU as compared to conventional practice (CTWOR-SSD-N120). Interestingly, root water uptake of CA+ at N rate of 150 kg N ha^−1^ (PBWR-SSD-N150) was 48% over CT (CTWOR-SSD-N120). The additional 30 kg N ha^−1^ in CA+ increased CRWU by 23% (PBWR-SSD-N150 vs. PBWR-SSD-N120).

#### Deep drainage

Adoption of SSDI leads to a reduction in deep drainage (DD) loss. Among the treatments, PBWR-SSD-N150 showed the lowest deep drainage (DD) loss, while the highest value was recorded for PBWR-Furrow-N120 (Table 2). The CA+ treatment PBWR-SSD-N120 and PBWR-SSD-N150 recorded 80 and 100 mm less DD as compared to the average of CA and CT, respectively (Table 1). Moreover, nearly 9% less DD loss was observed with an additional 30 kg N application ha^−1^ in PBWR-SSD-N150 over PBWR-SSD-N120. The largest fraction of applied water was lost as DD in CA-based plots, i.e., PBWR-Furrow-N120 plots (56%) and CTWOR-Furrow-N120 (54%) (Table 2). Total water lost by DD from CA+ plots was 21% less than FI plots (CTWOR-Furrow-N120, PBWR-Furrow-N120).

**Table 2:** Cumulative inflow and water-saving under different tillage, residue, irrigation and nitrogen management treatments.

Treatments^a^	Crop duration (113 days)		
	Irrigation water applied (mm)	Irrigation water saving (%)	
Plots with furrow irrigation	240	–	
Plots with subsurface drip irrigation	140	41.6	

Treatments	Cumulative inflow (mm)	Total water loss (%)	
PBWOR-SSD-N0	491.2	49.88	
PBWR-SSD-N0	491.2	46.45	
PBWOR-SSD-N120	491.2	47.29	
PBWR-SSD-N120	491.2	45.09	
PBWOR-SSD-N150	491.2	45.76	
PBWR-SSD-150N	491.2	40.94	
CTWOR-Furrow-N120	551.2	54.06	
PBWR-Furrow-N120	551.2	55.88	

^a^For treatment detail please see Table 4.

### Variation in soil water balance components and irrigation water saving

**Figure 5:**
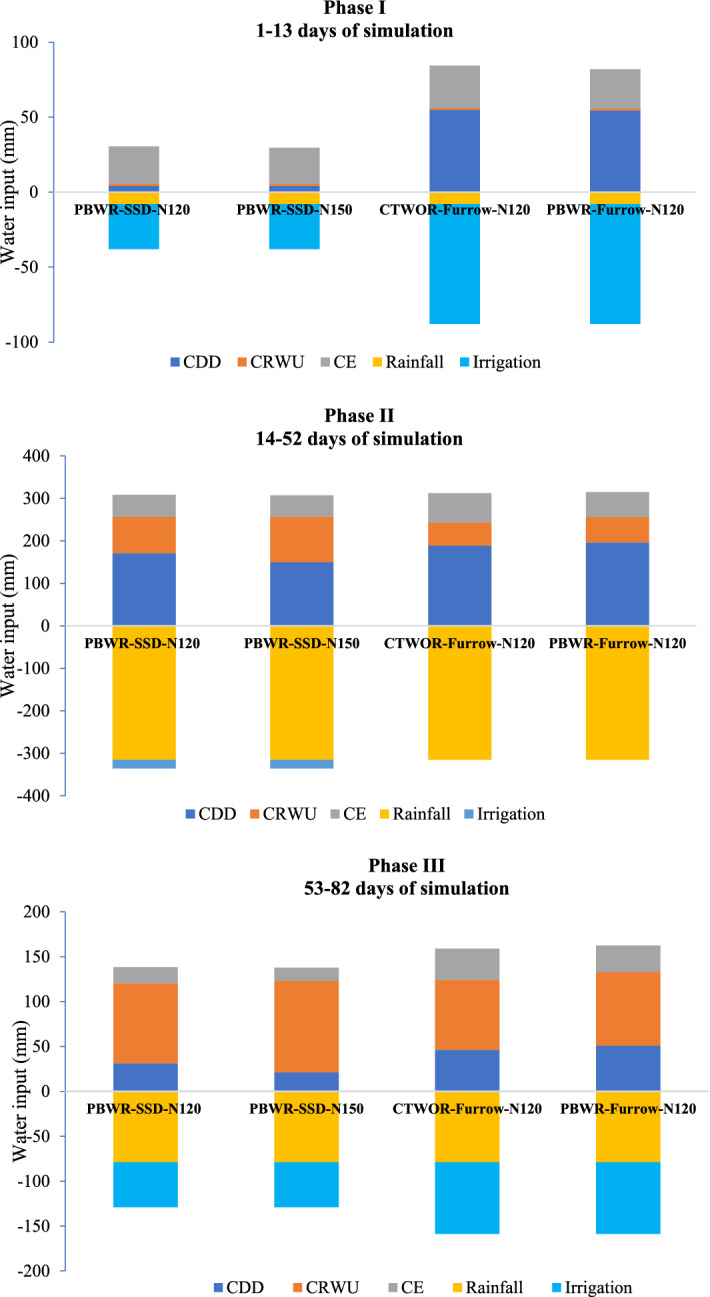
Partitioning of water input and output components in maize under contrasting tillage, residue, N and irrigation management treatments. Where, CDD: cumulative deep drainage; CRWU: cumulative root water uptake; CE: cumulative evaporation. Units of all parametersare in mm. *For treatment detail please see Table 4.

In the current study, we have divided the entire simulation period into three phases (Phase-I, Phase-II, Phase-III) to correlate/justify the variation of water input–output relation and crop growth over time (Fig. 5). For in-depth analysis we have selected two CA+, one CA alone and one conventional treatment viz., CA+ (PBWR-SSD-N120, PBWR-SSD-N150), CA (PBWR-Furrow-N120) and Conventional (CTWOR-Furrow-N120). Phase-I ranges from 1 to 13 DOS (8–20 DAS) corresponding to low rainfall and low canopy coverage, phase-II from14 to 52 DOS (21–59 DAS) with very high rainfall and moderate growth rate and Phase-III from 53 to 82 DOS (60–80 DAS) corresponding to moderate rainfall and peak crop growth rate. The rainfall received during Phase-I, phase-II and phase-III were 8, 321 and 73 mm, respectively.

In phase-I, the CRWU was very low in all treatments and the evaporation and DD components dominate as the sink of applied water. During this phase, FI-based plots (PB-Furrow-N120 and CT-Furrow-N120) received more than 50 mm of irrigation but that did not bring about any significant change to CRWU. The CRWU in the four treatments varied between 1.2 to 1.4 mm. The CA+ plots recorded an average 93% less DD signifying a huge potential of irrigation water saving during the initial crop phase when demand is very less. Irrespective of irrigation methods, in phase-I almost equal cumulative evaporation (CE) was recorded from all the treatments. In phase-II and III, average CRWU from SSDI-based plots were 96.7 mm and 95.5 mm, respectively, which amounts 70% and 20% over that of FI-based plots (CA alone and CT treatment). During phase-II and III, the CA+ (PBWR-SSD-N120) had 12% and 40% less cumulative evaporation, compared to CA plots, respectively. Increasing the N amount from 120 to 150 kg ha^−1^ in the CA+ plots curtailed additional 3.0% and 10% cumulative evaporation loss during phase-II and Phase-III, while increasing the CRWU by 25% and 14.2%, respectively. Maximum CRWU was recorded during Phase-III (60–80 DAS) which was 89.2, 101.9, 81.9 and 78.9 mm for PBWR-SSD-N120, PBWR-SSD-N150, PBWR-Furrow-N120 and CTWOR-Furrow-N120 plots, respectively. Adoption of SSDI led to ~ 62% and ~ 38% irrigation water saving during phase-I and phase-III, respectively, over conventional FI. Overall, in SSDI plots 41.6% irrigation water saving was recorded (on application basis) over conventional FI in maize crop (Table 2).

### Soil water content

**Figure 6:**
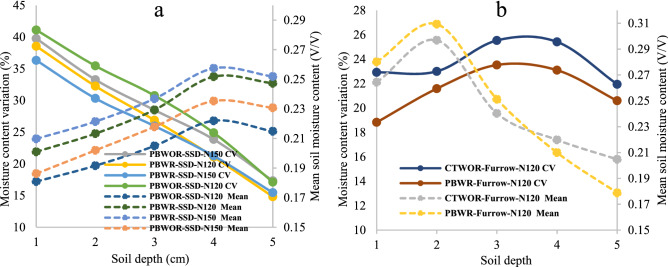
Variability in soil moisture content (primary axis) and mean moisture content (V/V) (secondary axis) of treatments with SSD and furrow irrigation during simulation period (8–89 DAS). Soil depth 1,2,3,4 and 5 corresponds to 0–5 cm, 5–10 cm, 10–15 cm, 15–30 cm and 30–45 cm soil depths, respectively. For treatment details please see Table 4.

Depth-wise mean moisture content and variation in soil moisture during the simulation period is depicted in Fig. 6. In SSDI plots, mean moisture content was lowest at the surface (0–5 cm) soil depth, then showed an increasing trend up to 15–30 cm (the zone where the laterals were laid in) and again decreased at 30–45 cm depth, indicating a reduction of both evaporation and DD loss. Across depth, mean soil moisture content in the CA+-based PBWR-SSD-N150 plots ranged from ~ 21% to 25% (Fig. 6a), whereas in the case of CT it ranged from ~ 20% to 29% (Fig. 6b). In FI-based treatment, the highest mean moisture content (30.9% and 29.7% for CA and CT respectively) was observed at 5–10 cm soil depth followed by surface 0–5 cm soil depth. In SSDI plots, the highest variations (CV) in moisture content (36–41%) was at surface soil and gradually decreased along with the depth, whereas an opposite trend was observed in furrow-irrigated plots (Fig. 6a,b). Overall, variations in moisture content throughout the soil profile were least in CA treatment (PBWR-Furrow-N120) (19% to 21%) followed by CT (CTWOR-Furrow-N120) (22 to 23%). The minimum soil moisture variability in CA and CT plots was observed at 0–5 cm soil depth followed by 5–10 cm depth. However, the soil moisture variability of CA+ plots was found to be dropped by 11.2, 12.3 and 27.3% for 10–15, 15–30 and 30–45 cm soil depths, respectively, as compared to average values of corresponding depths of FI plots (Fig. 6a,b).

### Radiation use efficiency, crop biomass yield and water productivity

Radiation use efficiency (RUE) is a function of N application rate. A similar RUE was recorded for all plots received sufficient rate of N (120 and 150 kg N ha^−1^), irrespective of tillage, irrigation and residue management options (Table 3). Moreover, treatments without N application showed significantly lower RUE as compared to N-fertilized treatments (Table 3). During the simulation period, the highest biomass accumulation was recorded for PBWR-SSD-N150 plots (19,260 kg ha^−1^) (Table 3). All N-fertilized CA+ treatments had a significantly higher dry matter accumulation as compared to FI-based plots (CTWOR-Furrow-N120 and PBWR-Furrow-N120). At a similar N-rate, the biomass accumulated in the CA+-based PBWR-SSD-N120 was ~ 20% and 16% higher than that of CT and CA plots, respectively.

**Table 3:** Dry matter accumulation, radiation use efficiency (RUE) and water productivity of maize under different tillage, crop residue, nitrogen and irrigation management treatments during the simulation period (8–89 DAS).

Treatments^a^	Dry matter accumulation (kg ha^−1^)	RUE (DM basis) (g MJ^−1^)	Water productivity (kg ha^−1^ mm^−1^)	
			$Crop\, WP:\frac{DM}{{\left( {{\text{CRWU}} + {\text{CE}}} \right)}}$	$Field \,WP:\frac{DM}{{Water inflow}}$
PBWOR-SSD-N0	3770d	2.04b	14.41d	7.68d
PBWR-SSD-N0	4145d	2.11b	15.75d	8.44d
PBWOR-SSD-N120	16686b	4.75a	62.50ab	33.98b
PBWR-SSD-N120	16906b	4.78a	62.20ab	34.42b
PBWOR-SSD-N150	17497ab	4.89a	63.74a	35.63ab
PBWR-SSD-N150	19260a	4.93a	65.53a	39.22a
CTWOR-Furrow-N120	14031c	4.50a	51.08c	25.41c
PBWR-Furrow-N120	14639c	4.57a	55.85bc	26.51c

Means followed by different lowercase letter within each column are significantly different (at P < 0.05) according to least significant difference test.

^a^For treatment detail please see Table 4.

In this study, the water productivity (WP) was estimated based on two different aspects and is presented in Table 3. Based on total water use (rainfall and irrigation), the highest WP (39.2 kg ha^−1^ mm^−1^) was observed for PBWR-SSD-N150 treatment which was at par with PBWOR-SSD-N150 and significantly higher than rest of the treatments. On the basis of evapo-transpiration, the WP of different treatments followed a similar pattern as of water use (rainfall and irrigation) basis. Despite the application of an equal amount of N doses, the WP (based on evapo-transpiration ET; CRWU + CE) of CA+ (PBWR-SSD-N120) was 11.4 and 21.7% higher than CA and CT treatment.

## Discussion

The challenge of water scarcity is increasing and becoming complex overtime, therefore, it is highly imperative to think beyond CA and to couple it with best-fit technologies, i.e., precision N and water application through SSDI (CA +) to minimize water loss, while increasing productivity, profitability and maintaining soil health.

### Soil water balance

The lower evaporation from CA-based plots was because of insulation properties of crop residues against solar radiation^35^, therefore allowing less energy flux into the soil, and decrease soil temperature^36^ and evaporation from the soil surface^37,38^. Crop residue also impeded water vapour diffusion and reduces wind speed at the soil-atmosphere interface^39^. Moreover, better plant growth was observed in CA+ treatments due to efficient water and N management, with simultaneous increase in radiation interception and decrease in evaporation. More importantly, in CA+ (CA and SSDI) avoiding wetting the soil surface (0–5 cm) through direct water application into the root zone at a depth of 15–20 cm led to a significant reduction in evaporation losses (~ 24–36% decrease in evaporation over CT). Similar to our results, Evett et al*.*^40^ reported lower soil evaporation under SSDI compared to FI. In the current study, despite receiving 100 mm of higher irrigation water over SSDI (during the entire season), the FI plots showed higher variability in soil moisture content in the root zone, which could be due to the higher DD and evaporation as compared to SSDI plots.

Adoption of CA+ not only reduced evaporation but also decreased DD losses. The highest DD loss from FI plots (Table 1), could be due to the application of a higher amount of irrigation water as compared to SSDI plots. Moreover, in FI system, the high-water infiltration into the soil from lateral (seepage) and vertical direction from a larger surface area (compared to SSDI, where the materials are below the soil surface), makes the soil saturated in a short period, thereby facilitating higher DD^9^. Patra et al*.*^41^ reported the formation of more water-conducting macropores, as well as biogenic pores in residue retained long-term PB plots, which make the soil more amiable for DD loss. In contrast, under CT condition soil structure and water-conducting pores were destroyed due to repetitive and intensive tillage operations^42^, which resulted in slightly lower DD in CT compared to CA.

A positive integration between N-fertigation (up to 150 kg N ha^−1^) through SSDI and residue retention was observed for root water uptake (Table 1). Between CA and CT plots, the higher root water uptake under CA (PBWR-Furrow-N120) treatment might be due to the fact of surface residue retention, which added additional nutrients to the soil, improved soil structure and root aeration. Moreover, the partitioning of water towards root water uptake under SSDI plots was higher due to lesser DD and evaporation losses. Phase-wise speaking, the higher DD losses in furrow irrigated plots during phase-I (1–13 DOS) was mainly due to the application of 50 mm more irrigation water than SSDI plots (Fig. 5). During phase-I, the root water uptake was very low, because crop canopy coverage and root growth were almost negligible, thus most of the applied water was lost by evaporation and DD. Therefore, precise irrigation water application through SSDI during phase I saved crop from water stress and aided to maintain turgidity, normal cell functioning without substantial losses. As crop proceeded towards phase II and phase-III, the transpiration dominated over evaporation and thus curtailed overall water losses. So, phase-II and III of CA+ can be summarized as diversion of water more towards CRWU by reducing evaporation and DD through precise water management using SSDI system. The highest root water uptake was recorded at Phase-III (between 60–80 DAS) (Fig. 5). Similar to Hydrus-2D model simulated finding, the highest consumptive use of maize at 55–82 DAS was also reported by Zegada-Lizarazu et al*.*^43^.

### Soil moisture variability

The decreased soil moisture variability in the root zone under CA+ was due to the controlled and frequent water application. However, in CT plots, the higher variability in soil moisture along the soil profile could be due to reduced water-stable aggregates, which gets further disrupted during drying and wetting tillage practices^44^. Thus, irrespective of the amount of water applied through furrow and SSDI methods, CA+ plots may act as a buffer for change in soil moisture content within the root zone.

Across the treatments, the mean moisture content at almost all depths was higher for SSDI irrigated plots when crop residue was retained (PBWR-SSD-N120 and PBWR-SSD-N150). The line source drip laterals were laid at 20 cm soil depth, which resulted in higher mean moisture content with least variability in 15–30 cm soil depth of SSDI plots (Fig. 6a,b). Reduced moisture content in surface soil justifies reduced rate of surface evaporation from SSDI plots. Also, crops in SSDI plots withdrew water from subsurface soil depth and accumulated higher biomass compared to FI treatments. In contrast, in FI treatments, the highest mean moisture and lowest CV were observed at the second soil layer (5–10 cm, just adjacent to the surface 0–5 cm) (Fig. 6b). In the experiment less variability in moisture content was observed in CA plots compared to CT plots. Similar results also reported by Parihar et al.^9^ with a justification that the lower mean weight diameter of soil aggregates in CT plots resulted in more unregulated sorption and desorption pattern of soil.

### Plant growth, RUE, LAI and WP

CA+ practices avoid extreme moist and dry conditions and help in maintaining optimum soil moisture content in the effective root zone. This facilitates higher root water uptake and crop growth, better canopy structure, higher leaf area index (LAI) in CA+ and ultimately resulted in higher light interception^45^. Moreover, N-fertigation through SSDI saves a considerable amount of N from leaching losses and a larger fraction of NO_3_-N remains readily available in the root zone for crop uptake^46^ Further, in the present study, irrigation was scheduled based on soil matric potential (below. − 50 kPa) in all CA+ plots, whereas it was based on critical crop growth stages in the case of CA and CT plots. Consequently, under FI plots, the high DD losses might have led to less N-availability due to potential NO_3_ leaching and slight water deficit in between two subsequent irrigations, therefore may cause reduction in plant growth. Higher LAI values of maize in no-tillage with residue retained condition was also reported by Bergamaschi et al*.*^47^.

The high dry-matter accumulation in CA+ plots, in particular, PBWR-SSD-N150 treatment could be due to precise and sufficient N and water application, in addition to residue retention. Interestingly, at the same N amount (120 kg ha^−1^) the higher (2.26 t ha^−1^) biomass accumulation of PBWR-SSD-N120 over PBWR-Furrow-N120 was solely due to efficient N and water management through SSDI. The 0.6 t ha^−1^ higher biomass accumulation in PBWR-Furrow-N120 over CTWOR-Furrow-N120, could be due to residue retention- and least soil disturbance across years (since 2014). The water productivity (WP) of different treatments also followed a similar trend as biomass accumulation (Table 3). The high WP of N-fertilized SSDI is mainly due to reduced water losses, while producing significantly higher dry biomass compared to CA and CT treatments. Despite the application of 60 mm more water during simulation period, proportionally lesser biomass was produced per unit of applied water under FI treatments. Among FI treatments, higher WP was observed in CA than that of CT. In agreement with the present findings, Parihar et al*.*^48^ also reported higher WP in CA-based PB systems as compared to CT. Results of the current study showed that CA+ treatments is more efficient in converting captured solar radiation into biomass thereby, recorded higher radiation use efficiency (RUE) (Table 3). While, lower RUE in FI plots was mainly due to poor canopy development which resulted in less radiation interception. Furthermore, RUE is a function of LAI^49^ and as discussed earlier, higher LAI was recorded under SSDI. A water-saving of 41.6% was achieved under CA+ over CA and CT. In a study Lamm and Trooien^50^ were also reported 35–55% saving of irrigation water under SSDI over FI. The reduction in irrigation requirement in SSDI plots was mainly because of reduced soil evaporation, decreased deep percolation and elimination of runoff losses^51^. Overall, in the present study, SSDI coupled with CA-practices at sufficient N application resulted in the highest moisture content, lowest variability the most effective root-zone layer (15–30 cm soil depth). Lower evaporations and DD losses, led to a higher cumulative root-water uptake, higher biomass accumulation, and ultimately higher WP. Despite the encouraging/promising results, further long-term studies should be conducted.

## Conclusions

Our research investigation has provided the *first science-based evidence* (using Hydrus-2D) on basic and in-depth scientific insights on soil water balance components for irrigation water saving, improving water productivity, radiation use efficiency and crop biomass yield under concurrent use of subsurface drip irrigation (SSDI) and conservation agriculture (CA) in maize grown in rotation with wheat. The results showed that bundling of complementing agronomic innovations like conservation agriculture (CA) coupled with subsurface drip irrigation (SSDI) and fertigation can be a sustainable option to address the emerging issues of water scarcity, declining groundwater table and shrinking and degraded natural resources. There is a huge potential for water savings in CA+ by minimizing the deep drainage and evaporation losses and potentially diverting the soil moisture use for consumptive uses. The healthy soil as a result of CA and CA+ favours more crop growth in terms of leaf area production, light interception and biomass production. So CA+ (CA + SSDI) is a better option/practice for irrigation water saving, soil moisture conservation, precision irrigation and nutrient management. Retaining crop residues on the soil surface as mulch is an alternative to in-situ crop residue burning. Besides, reduction in denitrification and volatilization losses through fertigation using SSDI system in CA will also help in reducing global warming potential in the long-run. Considering the future consequences of rapidly depleting groundwater resources, the provincial Governments in Punjab and Haryana as well as the Government of India have initiated a new policy program (“more crop per drop”, Water is Life, Direct Benefit Transfer of Electricity, etc.) for saving of water in agriculture. But there is a need for ‘Science driven Policy’ for effective execution of these investment schemes of the Government. Therefore, the results of our study complementing agronomic innovations i.e., CA and SSDI (CA+) for cereal crops would be of immense interest to farmers, policy planners and civil society for addressing the current and future challenges of farming. In the future the N dynamics and N loss studies in SSDI installed CA-based systems (CA+) using Hydrus-2D model may provide further insight into this aspect.

## Methods

### Experimental site

**Figure 7:**
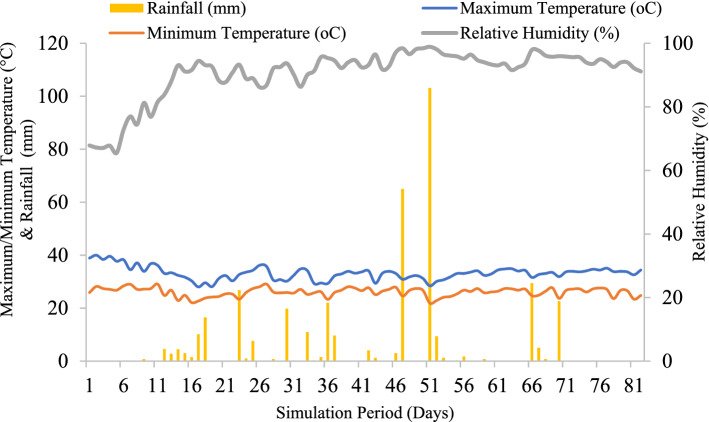
Daily weather conditions during simulation period (Day 1 corresponds to 28th June, 2019 and the day 82 corresponds to 17th Sep, 2019).

A field experiment was conducted in an ongoing research trial since *kharif-*2014 with the same set of treatments at fixed-site at Borlaug Institute for South Asia (BISA)-International Maize and Wheat Improvement Center (CIMMYT), Ladhowal (30.99°N latitude, 75.44°E longitude, 229 m above mean sea level), Punjab, India. The experimental site comes under Trans-Gangetic Plains Zone (Agro Climatic Zone-VI) of India with a sub-tropical and semi-arid climate. The average annual rainfall of the site is 734 mm with hot dry summer, wet monsoon and cold winters. The site receives about 80% of its total rainfall during the monsoon season. The total amount of rainfall received during the crop growing period of *Kharif-*2019 was 487.95 mm of which 82% was received during the simulation period (28th June to 17th September, 2019). The graphical representation of daily rainfall distribution, minimum and maximum temperature and relative humidity during simulation period have been presented in Fig. 7. The soil of the study site is of alluvial origin and well-drained, sandy loam in nature with pH range of 7.9–8.5 (1:2 soil water ratio) and EC 0.58 dS m^−1^.

### Experimental design and details

**Figure 8:**
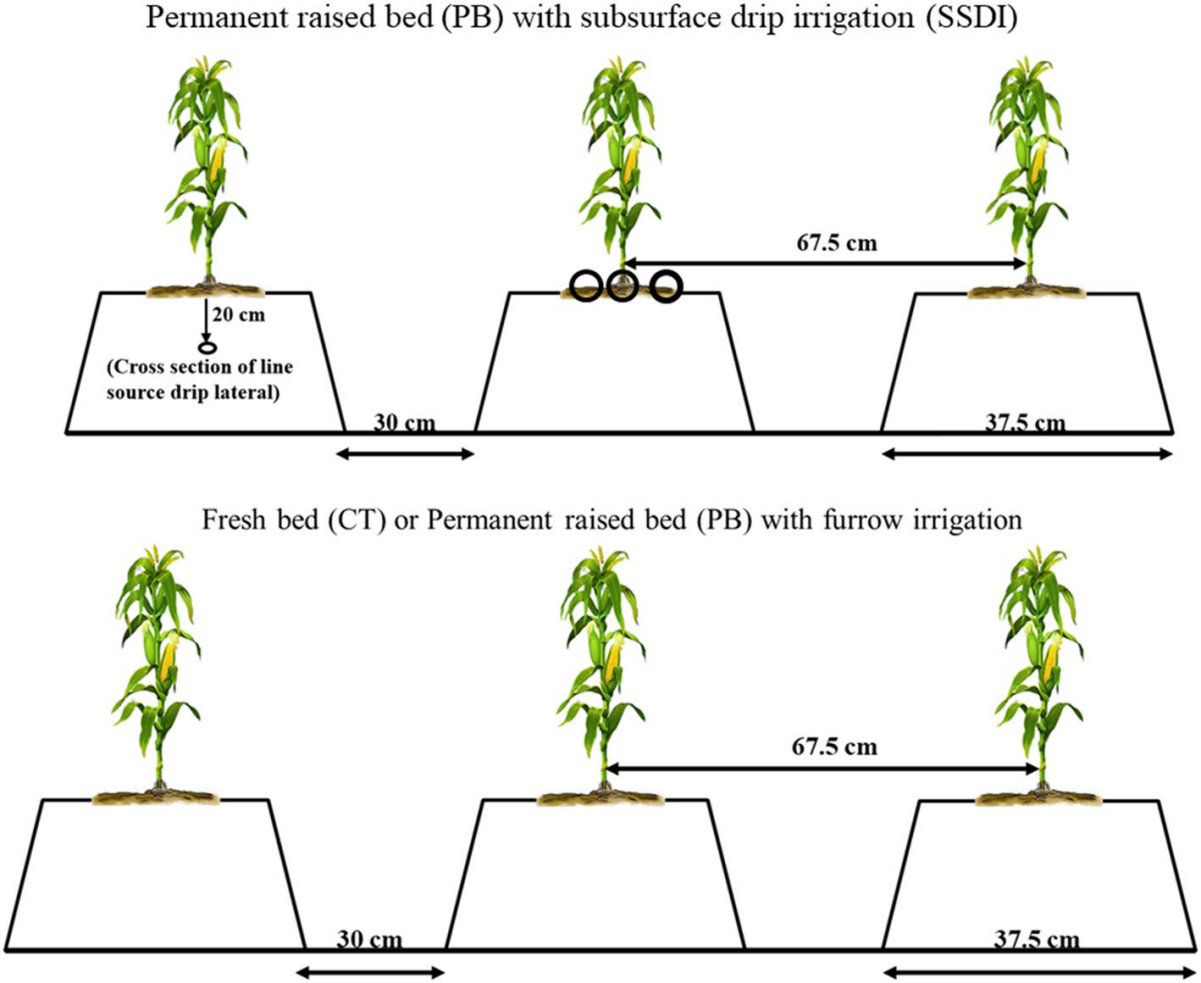
Land configuration and root sampling design (presented by circles) for two contrasting tillage and irrigation management options.

In this study, we assessed water budgeting in conservation agriculture-based sub-surface drip irrigation (CA+), CA and conventional practices (CT) using HYDRUS-2D for maize crop in 2019 (after five years of continued CA, CA+ and CT practices). Combinations of contrasting tillage (permanent beds-PB, and conventional tillage-CT), residue management (residue retention-WR and residue removal-WOR), irrigation methods (subsurface drip irrigation-SSDI and conventional furrow irrigation-FI) at different rates of nitrogen were adopted and SWB, water-saving, dry matter accumulation and water productivity were determined. The experiment was laid out in a randomized complete block design (RCBD) (plot size: 81 m^2^) using three replications. In the current study, 6 CA^+^ treatments (CA coupled with SSDI); permanent beds using sub-surface drip (PB-SSD) with and without crop residue at different N rates, viz*.*, 0, 120 and 150 kg N ha^−1^ were compared with CA (PB using furrow irrigation-FI with crop residue-120 kg N ha^−1^) and conventional practices (conventional tillage-CT using FI without crop residue-120 kg N ha^−1^) resulting in 8 treatments. The detail of treatments is given in Table 4. In all the WOR plots, 100% of crop residue of both the crops was removed, while in all WR plots, ~ 25% of preceding crop residue was retained on the soil surface since 2014. The permanent beds (PB) were prepared in June 2014 using a bed planter and were kept undisturbed across years with little reshaping once in a year. Bed width (mid-furrow to mid-furrow), width of flat tops and furrow depth were 67.5, 37 and 15 cm, respectively. A pictorial representation of land configuration is given in Fig. 8. In CT plots, land was prepared with one deep tillage followed by 2 ploughings with cultivators and fresh beds were prepared by bed maker. Across years, in all plots, maize variety P3396 was planted in June, at 67.5 cm × 20 cm spacing using maize planter. A common dose of 60 kg P_2_O_5_ + 30 kg K_2_O and 25 kg ZNSO_4_ heptahydrate ha^−1^ was applied to all plots as basal (directly at the time of mechanical seeding). N-fertilized treatments (PBWOR-SSD-N120, PBWR-SSD-N120, PBWOR-SSD-N150, PBWR-SSD-N150, CTWOR-Furrow-N120 and PBWR-Furrow-N120) received 23.5 kg N ha^−1^ along with the common basal dose of P_2_O_5_ and K_2_O. The rest of N in SSDI plots (PBWOR-SSD-N120, PBWR-SSD-N120, PBWOR-SSD-N150 and PBWR-SSD-N150) was applied through fertigation in 4-equal splits at 15 days interval (starting from 21 days after sowing-DAS). While in FI-plots (CTWOR-Furrow-N120 and PBWR-Furrow-N120) the remaining N was top-dressed into two equal splits at the knee-high stage and pre tasselling stage of maize. In SSDI plots irrigation was scheduled based on soil matric potential using a tensiometer (IRROMETER, River-side, California), installed at 20 cm depth at the middle (in between two rows) of each plot. Plots were irrigated at a soil matric potential of − 50 kPa throughout the entire crop period. During the entire crop period, for SSDI plots, 140 mm water was applied through 14-irrigations, with irrigation of 10 mm each. While in furrow irrigated plots (CTWOR-Furrow-N120 and PBWR-Furrow-N120) 240 mm irrigation water was applied in 6 irrigations. Out of the 6-irrigations in furrow-irrigated plots, two irrigations were given just after sowing and post-sowing (5-DAS), while the rest four irrigations were scheduled at 6 leaf, late knee-high, 50% silking and dough stages^52^. In SSDI plots, the frequency of irrigation varied 2–5 days according to the growth stage, weather conditions and rainfall event. In all the SSDI plots the laterals of drip were placed at 20 cm soil depth at a spacing of 67.5 cm. The inner diameter of laterals was 16 mm and were laid parallel to crop rows. Each lateral consisted of a line source emitter with a uniform emitter discharge of 2.0 L h^−1^ and were spaced at 30 cm. More details about these automated subsurface drip-irrigated systems (installed at CIMMYT-BISA Farm, Ladhowal) can be obtained from Sidhu et al*.*^17^.

**Table 4:** Treatment details and notation used in the study.

Tillage and residue management options	Irrigation methods	Nitrogen applied (kg ha^−1^)	Treatment notation used
Permanent Bed without residue	SSDI	0	PBWOR-SSD-N0
Permanent Bed with 25% wheat residue	SSDI	0	PBWR-SSD-N0
Permanent Bed without residue	SSDI	120	PBWOR-SSD-N120
Permanent Bed with 25% wheat residue	SSDI	120	PBWR-SSD-N120
Permanent Bed without residue	SSDI	150	PBWOR-SSD-N150
Permanent Bed with 25% wheat residue	SSD	150	PBWR-SSD-N150
Conventional tillage (fresh bed without residue)	FI	120	CTWOR-Furrow-N120
Permanent Bed with 25% wheat residue	FI	120	PBWR-Furrow-N120

SSDI: subsurface drip irrigation; FI: furrow irrigation.

### Simulation period

The long term experiment had started in *kharif-*2014. The data for model simulation in maize crop was collected during 2019. In this experiment, we have simulated profile soil moisture distribution, soil water balance and crop root water uptake for 82 days during 8-DAS to 89-DAS of maize crop. The initial seven days, two irrigations in furrow irrigated plots and four in SSDI plots (0–8 DAS) were not considered for simulation. Such irrigations were given to ensure uniform germination in extremely hot and dry weather conditions. Therefore, the plant hardly uptake any moisture from these irrigations. Throughout the simulation period (82 days; 8th–89th DAS) a total of 11 observations from 5 consecutive soil depths (0–5, 5–10, 10–15, 15–30 and 30–45 cm) were taken at 8 days intervals. The model calibration and Van Genuchten–Mualem parameter optimization (K*sa*t, θr, θs, α and n) procedure were performed using 30 data points (5 depths × 6 times of observation) in inverse solution technique and validated against 25 data point (5 depths × 5 times of observations). The model solves the water transport equation (Richards’ equation) in each mesh unit for both calibration and validation.

### Soil physical parameters measurement

For soil bulk density (BD) determination, before maize sowing (19th June 2019), undisturbed soil samples were collected from each plot in triplicate from soil depths of 0–5, 5–10, 10–15 and 15–30, 30–45 cm. The soil water content for the same soil depths was measured volumetrically (gravimetric moisture content × BD). Similarly, soil moisture content at saturation, field capacity and permanent wilting point was measured using pressure plate apparatus^53^. The soil texture parameters, i.e., sand, silt and clay percentages for the same depths were obtained using the Hydrometer method^54^.

### Root sampling and scanning

The procedure for root sampling was followed as described by Aggarwal et al*.*^55^. Root samples were taken from the field at 30 and 56 DAS using a root core sampler. Three representative plants from each plot were cut from the base and roots were taken from the center (0, 0) and 2 cm left and right from the periphery at 0–15, 15–30 and 30–45 cm soil depths. The collected samples were taken in a tray and soaked for 24 h to separate roots from the adhered soil. A pinch of sodium hexametaphosphate was added for quick dispersion of soil particles. The roots were washed thoroughly under tap water and passed through a series of sieves^9^. Thereafter, the washed roots were scanned using a rhizo-scanner and analyzed using Winrhizo software scanner (Regent Instruments Inc., Canada). The root length density (RLD) was obtained from the following formula:

1 $$ {\text{RLD}} = \frac{{{\text{Total root length}}\;\left( {{\text{cm}}} \right)}}{{{\text{Volume of the root sampling auger}}\;\left({{\text{cm}}^{3} } \right)}} $$

The depth of maximum rooting was fixed up to the point until the RLD value approached 0.05 cm cm^−3^).

### Calculation of fractional intercepted photosynthetically active radiation (fIPAR)

In this experiment, line quantum sensor LI-191SA (LICOR Inc., Lin 184 coln, NE, USA) was used to measure incoming and outgoing photosynthetically active radiation (PAR) at the top as well as bottom of the canopy at 7-days interval. Fractional intercepted PAR (fIPAR) was calculated using the following formula:

2 $$ {\text{fIPAR}} = \frac{{{\text{I}}_{{\text{o}}} - {\text{I}}_{{\text{t}}} }}{{{\text{I}}_{{\text{o}}} }} $$

where I_o_ and I_t_ are incident and transmitted PAR at the top and bottom of the canopy respectively. The daily values for the whole simulation period were finally calculated by the linear interpolation method. Measured values of fIPAR at various crop growth stages in different treatments are shown in Fig. 1.

### Partitioning of evaporation and transpiration component

For simulating soil moisture distribution, soil water balance and root water uptake using the Hydrus-2D model, potential maize evaporation and transpiration values were supplied to the model separately. To calculate potential crop evapotranspiration (ETc), reference evapotranspiration (ETo) is a prerequisite. Here, ETo was calculated using FAO-CROPWAT 8.0 model^56^. Etc was calculated by multiplying ETo crop coefficient. The partitioning of ETc into evaporation (Ep) and transpiration (Tp) was achieved by the following equation as described by Ritchie^57^:

3 $$ {\text{E}}_{{\text{p}}} = {\text{ETc}}\exp { }\left( { - \kappa {\text{LAI}}} \right) $$

where κ is the canopy radiation extinction coefficient and LAI is leaf area index by calculating the negative slope of the relationship between ln (1 − fIPAR) and LAI with intercept set to zero using the following equation^9^:

4 $$ {\text{k}} = \frac{{{\text{ln}}\left( {1 - {\text{fIPAR}}} \right)}}{{{\text{LAI}}}} $$

### The Hydrus-2D model

Here, the Hydrus-2D model was used to simulate the spatio-temporal variation of soil water balance components (evaporation, root water uptake, deep drainage, soil water content). The model assumes soil as a porous medium with a homogeneous isotropic structure and neglects the effect of temperature and gas on soil water movement. The governing principle behind the two-dimensional water movement in soil lies in Richard’s equation^58^:

5 $$ \frac{\partial \theta }{{\partial t}} = \frac{\partial }{\partial x}\left[ {K\left( h \right)\frac{\partial h}{{\partial x}}} \right] + \frac{\partial }{\partial z}\left[ {K\left( h \right)\frac{\partial h}{{\partial z}}} \right] + \frac{\partial k \left( h \right)}{{\partial z}} - S\left( {x,z,h} \right) $$

where θ is the volumetric water content (cm^3^cm^−3^); *h* is the pressure head of water in the soil matrix, *K(h)* is the unsaturated hydraulic conductivity (cm s^−1^) and *S (x, z, h)* is the root water uptake sink term (s^−1^). The sink term *S (x, z, h)* for root water uptake can be described by Feddes et al*.*^59^ equation:

6 $$ S\left( {\psi ,x,z} \right) = \alpha_{s} \left( {\psi ,x,z} \right) \times b\left( {x,z} \right) \times T_{p} \times L $$

where $\alpha_{{\text{s}}} \left( {\psi , {\text{x}}, {\text{z}}} \right):$ is the dimensionless function of soil water stress, 0 ≤ α (x, z, h) ≤ 1; b (x, z) is the normalized root water uptake distribution function (cm^−2^); W: Surface width associated with atmospheric boundary (cm); Tp: Potential rate of transpiration (cm day^−1^). More detailed information on this aspect can be found from Aggarwal et al*.*^23^.

### Domain geometries and discretization

**Figure 9:**
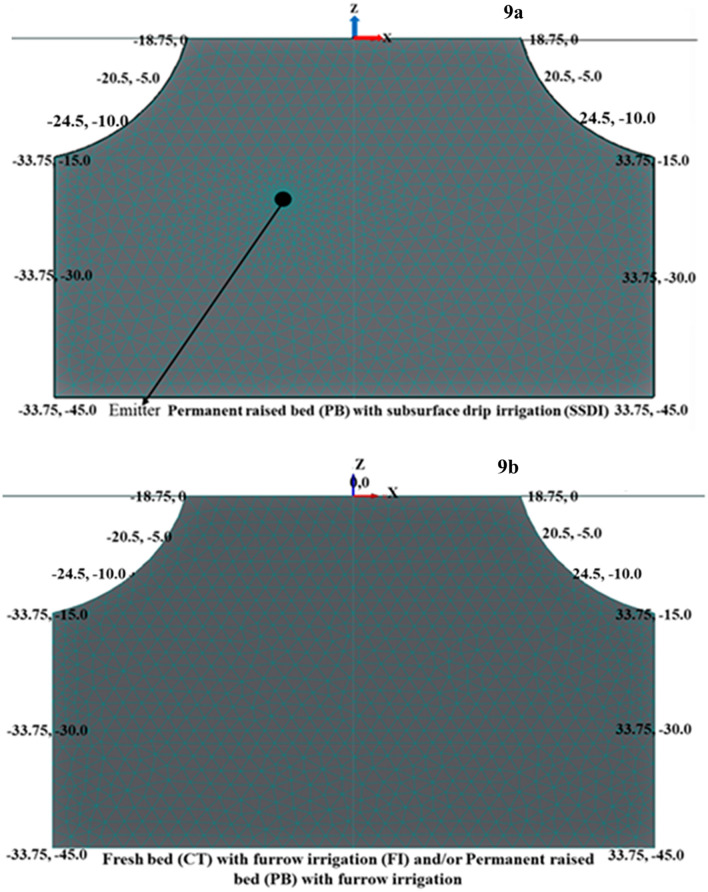
Geometries characterization of different land configuration.

Geometries depicting soil transport domain of 0–5, 5–10, 10–15 and 15–30, 30–45 cm soil depths are presented in Fig. 9. The model suggested default value (2.20 cm) was used as targeted finite element size for spatial discretization and mesh generation. The finite element size is 0.1 cm where emitters are located, and element size gradually increases with distance till approaches the default value. The node point of the plant base was assigned as (0, 0) and the coordinates of the upper boundary on the left and right side of the soil domain were kept at (− 18.75, 0) and (18.75, 0) points, respectively. Similarly, the lower boundary coordinates of the left and right sides were (− 33.75, − 45) and (33.75, − 45), respectively. Five observation nodes were inserted in transport domain at (0, – 2.5) (0, – 7.5), (0, – 12.5) (0, – 22.5) and (0, – 37.5) coordinates to obtain information about spatio-temporal soil water changes. Model-derived soil water content (V/V) at these observation points were compared with volumetrically obtained soil water content (SWC) at the base of the plant (at 0–5, 5–10, 10–15, 15–30 and 30–45 cm soil depths).

### Initial condition

The initial measured SWCs for all treatments at 0–5, 5–10, 10–15, 15–30 and 30–45 cm soil depths at beginning of the simulation (28th June) were taken as the input of model initial condition.

### Boundary condition

The upper and lower boundary of the soil domain was assigned as atmospheric boundary and free drainage boundary, respectively. The vertical sides of the domain have no flux boundaries.

### Calibration and validation

To simulate soil water distribution, root water uptake pattern, the Hydrus-2D model needs to be provided with van Genuchten–Mualem (VGM) parameters^60^, i.e., residual water content (θr), saturated water content (θs), saturated hydraulic conductivity (Ks), inverse of air entry suction value (α), pore connectivity parameter (l), and pore size distribution index (n). Among these parameters only α, n and *Ks* were generated using a neural network prediction program with Rosetta software package^61^. For these, the prediction program was supplied with soil texture and initial bulk BD inputs. The selected parameters were further optimized using the inverse optimization method (inverse solution) provided with 5 iterations. The detailed steps for optimization are described by Parihar et al*.*^9^ and Rai et al.^62^. In this present study total 55 data points were collected (combining 5 depth and 11 times of observation) for each treatment during the simulation period of crop growth (2019) and out of 55, 30 were used for calibration and the remaining 25 data points was used for validation. The soil moisture data (30 data points) at 1st, 8th, 32nd, 56th and 64th, 82nd (final) day of the simulation period were used in inverse solution for calibration of the model. For validating the accuracy of the model output, simulated values of SWC (V/V) from 5 observation nodes (0, – 2.5) (0, – 7.5), (0, – 12.5) (0, – 22.5) and (0, – 37.5) were compared with volumetrically calculated (near the base of plants) SWC of 0–5, 5–10, 10–15, 15–30, 30–45 cm soil depths.

### Soil water balance

Soil water balance of the soil profile (0–45 cm) during the simulation period (8–89 DAS) was described using the following equation:

7 $$ SWC_{initial} + Rain \left( {or, Irrig.} \right) = SWC_{final} + Cum.RWU + Cum.Evap + Cum.Drain $$

where S*WC* initial and final: Cumulative soil water content (mm) of 0–45 cm soil depth at start and end of the simulation period, respectively, *Rain*: Cumulative rainfall (mm) received during simulation period, *Irrig.*: Total irrigation amount (mm) applied during simulation period, *Cum. RWU:* Cumulative root water uptake (mm), *Cum. Evap*: Cumulative surface evaporation (mm), *Cum. Drain*: Cumulative deep drainage (mm).

### Model accuracy and performance analysis

The model accuracy and performance were evaluated under the following criteria: coefficient of determination (R^2^), model efficiency (ME) and Root Mean Square Error (RMSE). The R^2^ describes the strength of linear regression between simulated and observed SWC. Its value ranges between 0 (no correlation) to 1 (perfect correlation). The formula for determining R^2^ is:

8 $$ R^{2} = \frac{{\left( {\mathop \sum \nolimits_{i}^{n} \left( {O_{i} - O_{m} } \right)\left( {P_{i} - P_{m} } \right)} \right)^{2} }}{{\mathop \sum \nolimits_{i}^{n} (O_{i} - O_{m} )^{2} \times \mathop \sum \nolimits_{i}^{n} (P_{i} - P_{m} )^{2} }} $$

where *Oi*: Field observed moisture data and Pi: Model simulated moisture data. *Om* and *Pm* are the mean values. The model performance in terms of accuracy was measured by model efficiency (ME)^63^. The ME ranges between − ∞ to 1. A negative value indicates the poor performance of the model while any value closer to 1 indicates better efficiency. The following formula was used to describe ME:

9 $$ ME = 1 - \frac{{(\mathop \sum \nolimits_{i}^{n} \left( {O_{i} - P_{i} } \right)^{2} }}{{(\mathop \sum \nolimits_{i}^{n} \left( {O_{i} - O_{m} } \right)^{2} }} $$

The quantity of deviation between field observed SWC and model-simulated SWC can be described by RMSE in terms of the actual size of error and it was calculated as:

10 $$ RMSE = \sqrt {\frac{{\mathop \sum \nolimits_{i = 1}^{n} \left( {O_{i} - P_{i} } \right)^{2} }}{n}} $$

### Crop and field water productivity

Water productivity (WP) with respect to crop and field was calculated using the following formulas:

11 $$ Crop \;WP \left( {{\text{kg ha}}^{ - 1} {\text{mm}}^{ - 1} } \right) = \frac{{Crop \;biomass \;accumulated \;upto\; 89\; DAS\; \left( {{\text{kg ha}}^{ - 1} } \right)}}{{Root \;water \;uptake \;\left( {{\text{mm}}} \right) + Evaporation \;\left( {{\text{mm}}} \right)}} $$

12 $$ WP \;\left( {{\text{kg ha}}^{ - 1} {\text{mm}}^{ - 1} } \right) = \frac{{Crop\; biomass\; accumulated \;upto \;89 \;DAS \;\left( {{\text{kg ha}}^{ - 1} } \right)}}{{Rainfall\; \left( {{\text{mm}}} \right) + Irrigation\; \left( {{\text{mm}}} \right)}} $$

### Radiation use efficiency

Daily incoming solar radiation was computed from bright sunshine hours using the Angstorm equation^64^. Incident PAR was obtained by multiplication of daily incoming solar radiation with a factor of 0.48. Incident PAR was multiplied with corresponding linearly interpolated fIPAR values to obtain daily intercepted PAR (IPAR). Total IPAR (TIPAR) of the simulation period was obtained through the integration of daily IPAR. Radiation use efficiency (RUE) was calculated based upon the net biomass produced during the simulation period. RUE was obtained from the slope of the linear plot between biomass yield and TIPAR.

### Data analysis

The data on different parameters were subjected to the analysis of variance (ANOVA) for Randomized Block Design (RBD)^65^ using the general linear model procedure of the statistical analysis system (SAS Institute, Cary, NC). The differences between treatment means were performed by Fisher’s least significant difference (LSD) test at P < 0.05. The calculated values of coefficient of variation from simulated soil moisture content were used to express soil moisture variability of different layers.
